# Dissociation in Neural Correlates of Hyperactive/Impulsive vs. Inattentive Symptoms in Attention-Deficit/Hyperactivity Disorder

**DOI:** 10.3389/fnins.2022.893239

**Published:** 2022-06-22

**Authors:** Yu Luo, Jack H. Adamek, Deana Crocetti, Stewart H. Mostofsky, Joshua B. Ewen

**Affiliations:** ^1^Kennedy Krieger Institute, Baltimore, MD, United States; ^2^School of Biological Science and Medical Engineering, Beihang University, Beijing, China; ^3^Department of Neurology, Johns Hopkins University School of Medicine, Baltimore, MD, United States; ^4^Department of Psychiatry and Behavioral Sciences, Johns Hopkins University School of Medicine, Baltimore, MD, United States

**Keywords:** ADHD inattention, ADHD hyperactivity/impulsivity, interhemispheric connectivity, event-related desynchronization, motor execution

## Abstract

Attention-deficit/hyperactivity disorder (ADHD) is one of the most common neurodevelopmental disorders characterized in current diagnostic criteria by two dominant symptoms, inattention and hyperactivity/impulsivity. Here, we show that task-related alpha (8–12 Hz) interhemispheric connectivity changes, as assessed during a unimanual finger-tapping task, is correlated with inattentive symptom severity (*r* = 0.55, *p* = 0.01) but not with severity of hyperactive/impulsive symptoms. Prior published analyses of the same dataset have already show that alpha event-related desynchronization (ERD) in the hemisphere contralateral to unimanual tapping is related to hyperactive/impulsive symptom severity (*r* = 0.43, *p* = 0.04) but not to inattentive symptom severity. Our findings demonstrate a neurobiological dissociation in ADHD symptom severity, with implications for understanding the structure of endophenotypes in the disorder as well as for biomarker development.

## Introduction

Attention-deficit/hyperactivity disorder (ADHD) affects approximately 5% of children worldwide (Richarte et al., 2021), resulting in impairments in academic, occupational, family and social functioning (Demontis et al., 2019). Currently, ADHD is diagnosed based on criteria defined in the American Psychiatric Association’s Diagnostic and Statistical Manual (DSM-5) (American Psychiatric Association, 2013) or World Health Organization’s International Classification of Diseases (ICD-11), in which inattention and hyperactivity/impulsivity are core but independent ADHD symptom domains (Thapar and Cooper, 2016).

Under DSM-5, there are three explicit presentations of ADHD: predominantly inattentive (ADHD-I), predominantly hyperactive/impulsive (ADHD-H/I) and combined presentation (ADHD-C). Questions exist as to how inattentive and hyperactive/impulsive symptoms relate to each other. One possibility is that the two symptom clusters reflect different manifestations of the same biology. For example, the ADHD-H/I presentation is uncommon in school-age children (Lahey et al., 2005) and is most often seen in the preschool age group (O’neill et al., 2017). The reason that the ADHD-H/I presentation is seen in preschoolers yet transitions into ADHD-C is believed by some clinicians to reflect a change in social expectations, rather than a change in underlying biology: little sustained attention is expected from preschoolers and increasing attentional expectations as a child reaches school age uncover pre-existing (but yet-untested) limitations.

At the group level, the inattentive and hyperactive/impulsive symptoms of ADHD show a high degree of internal consistency, with correlation coefficients between 0.63 and 0.75 (Willcutt et al., 2012; Sokolova et al., 2016), and twin studies showing a strong shared genetic association (*r* = 0.90) (Greven et al., 2011; Kuntsi et al., 2014). Modeling of large datasets points toward a causal relationship between inattentive and hyperactive/impulsive symptoms (Willcutt et al., 2012; Sokolova et al., 2016). These observations suggest some shared underlying neurobiology, yet on the other hand, clinical evidence remains for the subtypes existing as distinct entities (Graetz et al., 2001). It therefore remains an open question as to whether inattentive and hyperactive/impulsive symptoms are different manifestations of the same brain mechanisms (Sanefuji et al., 2017) or represent a combined mechanism manifesting under different social expectations.

Neuroimaging offers a method for external validation of the structure of cognitive mechanisms (Poldrack and Yarkoni, 2016; Ewen et al., 2021). Clear dissociations in brain-behavior correlations offer support for considering two cognitive mechanisms to be distinct, even when the cumulative brain data do not offer a clear picture of the underlying brain mechanism itself. When it comes to assessing whether hyperactive/impulsive and inattentive symptoms of ADHD should be considered distinct based on separable brain responses, vs. different manifestations of the same underlying neurobiology, data are limited. One recent study showed a double dissociation in resting-state networks (Sanefuji et al., 2017) but the literature is otherwise sparse. The determination of biological dissociations across clinical phenomena is critical to the advancing current, early-stage efforts in biomarker development within neurodevelopmental disabilities and neuropsychiatry (Sahin et al., 2018; Ewen et al., 2019; Ewen and Levin, 2022).

Our group has recently used EEG to examine the relationship between brain oscillations and ADHD symptoms (Luo et al., 2022), finding that hyperactive/impulsive (but not inattentive) symptoms were associated with task-related alpha event-related desynchronization (ERD) in children with ADHD during sequential finger-tapping in the non-dominant hand (*r* = 0.43, *p* = 0.04) (Luo et al., 2022). In the present study, we explored the associations between interhemispheric connectivity measures and clinical ADHD symptoms, hypothesizing that we might again observe dissociations of electrophysiologic correlates of hyperactive/impulsive vs. inattentive symptom domains.

## Materials and Methods

### Participants and Clinical Assessment

The participants were identical to those reported in our previous studies (Mcauliffe et al., 2020; Luo et al., 2022). Fifty children (37 males, aged 8–12 years) participated in the study. Twenty-five (18 males; 8 children with ADHD-I and 17 children with ADHD-C) were diagnosed with ADHD using the Diagnostic Interview for Children and Adolescents, Fourth Edition (Reich, 2000) (DICA-IV) or the Kiddie Schedule for Affective Disorders and Schizophrenia (K-SADS) (Kaufman et al., 1997). Twenty-five (19 males) were age- and sex-matched typically developing (TD) controls. All participants were right-handed individuals without any history of other psychiatric or neurological disorders. Handedness was assessed by the Edinburgh Handedness Inventory (Oldfield, 1971). The study was approved by the institutional review board of Johns Hopkins Medicine, Baltimore, MD. Written informed consent and oral assent were obtained from the legal guardians and children, respectively, before the study.

The ADHD Rating Scale (Fabiano et al., 2006) was used to confirm the diagnosis. The Conner’s Rating Scale-Revised (CPRS-R) (Conners et al., 1998) was used to quantify Hyperactive/Impulsive (H/I) and Inattentive ADHD symptoms. All children with full-scale IQ (FSIQ) scores (Wechsler, 2014) below 80 were excluded from the study. Because children with ADHD tended to have a lower IQ than TD controls as assessed by the General Ability Index (GAI) (*p* = 0.062, Cohen’s *d* = 0.54), all analyses were adjusted for IQ.

### Study Design

Motor overflow was measured and quantified using the TSD131 finger twitch transducers (BIOPAC Systems Inc., Goleta, CA, United States). Participants were required to successively tap each finger against the thumb with self-paced timing in a fixed order (index-middle-ring-little), with alternating trials of left-handed (non-dominant) finger tapping (LHFT) and right-handed (dominant) tapping (RHFT). A start cue was given *via* a video monitor. There were five blocks consisting of 20 trials per block, and each trial lasted 6 s, with a one-second rest/baseline period prior to the “Go” cue in each trial. LHFT and RHFT trials alternated within block. The first trial was LHFT or RHFT randomly assigned among participants, and the number of trials of LHFT and RHFT were equal. Behavioral overflow was computed by averaging the cumulative angular deflection of the non-tapping hand for all trials across blocks together at the same time during LHFT and RHFT.

### EEG Data Acquisition and Analysis

**FIGURE 1:**
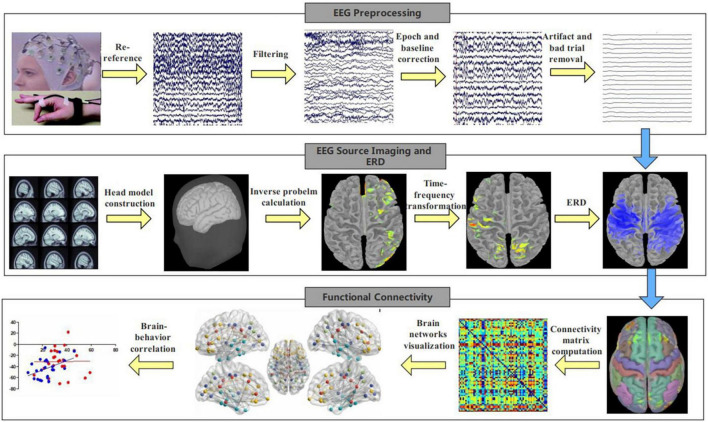
EEG analysis pipeline. EEG signals were recorded during finger-tapping task, and EEG analysis included preprocessing, EEG source imaging, ERD calculation and functional connectivity computation.

EEG data analysis pipeline is shown in Figure 1. EEG data were recorded using a 47-channel WaveGuard cap system and asa-lab amplifier. The sampling rate was 1,024 Hz; data were down-sampled to 512 Hz. For EEG preprocessing, we first removed non-cerebral electrodes (including electrooculogram), and then loaded in the channel localization file. Next, we filtered EEG data using a 1 and 80 Hz finite impulse response (FIR) filter. A 60 Hz notch filter was used to eliminate the line noise. EEG recordings were then segmented into epochs with lengths from −1 to 5 s with respect to the onset of the trial’s “Go” cue. Baseline correction was performed after segmentation. EEG data was re-referenced to the average of all electrodes. A blind source separation algorithm was used to identify and remove artifacts (Delorme et al., 2012). Finally, we interpolated channels with excessive artifact and removed trials with excessive artifact. There were no more than two channels excluded in any participant.

To examine the physiological changes underlying behavior, we performed EEG functional connectivity analyses on the source level using the Brainstorm toolbox (Tadel et al., 2011). First, we conducted EEG source-imaging analysis (Luo et al., 2019; Luo and Zhang, 2020) by computing the forward model using OpenMEEG and calculating the inverse problem using the standardized low-resolution brain electromagnetic tomography (sLORETA) algorithm (Pascual-Marqui, 2002). Second, we used the Morlet wavelet algorithm to compute ERD based on the EEG source imaging data as in Luo et al. (2022). Finally, we calculated the interhemispheric functional connectivity in the sensorimotor areas using amplitude envelope correlation (Samiee and Baillet, 2017) from source-level data. Functional connectivity was calculated from the average amplitude envelope correlation of the regions of interest (ROI) whose border was defined using the Brodmann atlas (Maldjian et al., 2003). The ROI included the somatosensory cortex, primary motor cortex, supplementary motor area and dorsal premotor cortex. The ROI was selected because previous studies suggest these regions were involved in motor execution (Ewen et al., 2016; Mcauliffe et al., 2020; Luo et al., 2022). Regarding the connectivity calculation, we first computed the “task” functional connectivity using the data from 1.5 to 3 s after movement onset, since mirror overflow was likely to occur during this time window (Mcauliffe et al., 2020). Then we calculated the “baseline” functional connectivity using the data from −1 to 0 s (baseline period) in each trial. Finally “baseline” was subtracted from “task” functional connectivity so that we exclude the influence of other factors besides the mirror overflow. We performed such connectivity analysis for each participant. We focused on connectivity during LHFT, as we did with ERD previously (Luo et al., 2022), because of observed behavioral group differences during LFHT and not RHFT.

### Statistical Analysis

We ran independent-samples *t*-tests between ADHD and TD groups to examine potential differences in demographics, behavioral mirror overflow (*via* goniometer) and clinical data (Conners ADHD Inattentive and Conners ADHD Hyperactive/Impulsive). All *t*-tests were two-sided, with α = 0.05. To examine the relationship between ADHD symptom severity and interhemispheric functional connectivity, we calculated Pearson’s *r* independently in the ADHD and TD group. GAI was used for IQ statistical adjustment, as is typical in ADHD literature, because it is designed to be insensitive to working memory and processing speed differences; these cognitive traits are considered to be key aspects of the ADHD phenotype. We also adjusted for age and sex in the statistical analyses.

## Results

### Demographics and Behavioral Results

As shown in Table 1 and as previously reported in Mcauliffe et al. (2020) and Luo et al. (2022), there were no significant differences in demographics, including age (*p* = 0.31, Cohen’s *d* = 0.29), sex (*p* = 0.75, Cohen’s = 0.09), and GAI (*p* = 0.06, Cohen’s *d* = 0.54) between the ADHD and TD groups. Moreover, there were no significant differences in the behavioral motor overflow during (dominant) RHFT (*p* = 0.25, Cohen’s *d* = 0.46). However, compared to TD controls, children with ADHD showed significantly more motor overflow during (non-dominant) LHFT, with a large effect size (*p* = 0.01, Cohen’s *d* = 0.88).

**TABLE 1:** Demographics and behavioral results between children with ADHD and TD controls.

	ADHD			TD	*t*-test results	
			
	ADHD total	ADHD-I	ADHD-C	TD total	*p*-value	Cohen’s *d*
*N*	25	8	17	25	–	–
Gender (Female/Male)	7/18	3/5	4/13	6/19	0.75	0.09
Age	10.36 (1.24)	10.64 (1.37)	10.22 (1.20)	10.73 (1.33)	0.31	0.29
General Ability Index (GAI)	112.48 (11.41)	110.38 (11.02)	113.47 (11.78)	118.72 (11.68)	0.06	0.54
LHFT overflow	30.64 (16.70)	25.52 (12.10)	33.05 (18.30)	17.57 (12.75)	0.01**	0.88
RHFT overflow	23.26 (14.70)	18.44 (15.42)	25.37 (14.35)	15.57 (18.61)	0.25	0.46

The behavioral results were adjusted for age, sex and GAI. ADHD denotes children with attention-deficit/hyperactivity disorder, ADHD-I denotes children with ADHD of the inattentive type, ADHD-C denotes children with ADHD of the combined type, and TD denotes typically developing controls. Data are presented in mean ± standard deviation (SD) formats. Student’s t-tests were performed between the combined ADHD cohort and the TD cohort. **Indicates p ≤ 0.01.

### Group Differences in Task-Related Functional Connectivity Modulation

Children with ADHD did not show decreased task-related changes in interhemispheric functional connectivity between the left and right sensorimotor areas in alpha during LHFT in comparison with TD controls, though the within-sample difference was of a moderate effect size (ADHD mean = 0.003 ± 0.06, TD mean = −0.02 ± 0.04, *p* = 0.14, Cohen’s *d* = 0.45).

### Correlations Between Alpha Event-Related Desynchronization and Functional Connectivity

**FIGURE 2:**
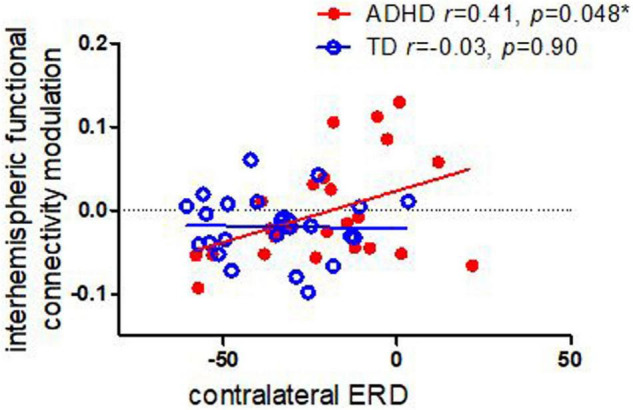
Correlations between task-related interhemispheric functional connectivity modulation in alpha and contralateral ERD in children with ADHD TD controls. Children with ADHD showed a significant association between functional connectivity modulation and ERD, whereas controls did not.

As shown in Figure 2, we found that, among children with ADHD, there was significant correlation between contralateral alpha ERD and interhemispheric functional connectivity (*r* = 0.41, *p* = 0.048); whereas there was no such significant correlation among TD controls (*r* = −0.03, *p* = 0.90). We did not find significant correlation between ipsilateral alpha ERD and functional connectivity in either children with ADHD or TD controls.

### Correlations Between Mirror Overflow and Functional Connectivity

Children with ADHD did not show a significant correlation between functional connectivity and mirror overflow (*r* = −0.10, *p* = 0.64), whereas there was a significant correlation between connectivity and overflow in TD controls (*r* = −0.50, *p* = 0.01).

### Correlations Between Attention-Deficit/Hyperactivity Disorder Symptoms and Functional Connectivity

**FIGURE 3:**
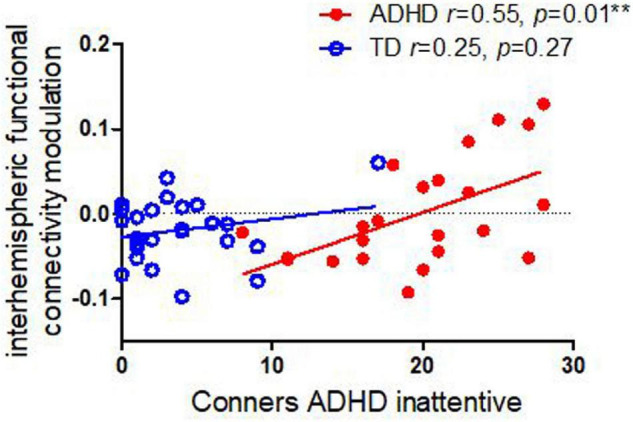
Correlations between interhemispheric functional connectivity modulation in alpha and Conners ADHD inattentive and H/I symptoms in children with ADHD and TD controls. Children with ADHD showed an association between inattentive symptom severity and task-related modulation of connectivity, whereas the TD cohort did not.

As shown in Figure 3, we identified that the interhemispheric functional connectivity of sensorimotor regions was correlated with Conners Inattentive symptoms in children with ADHD (*r* = 0.55, *p* = 0.01); this relationship persisted even when adjusting for contralateral ERD (*p* = 0.038). There were no significant correlations between interhemispheric functional connectivity and Conners inattentive in TD controls (*r* = 0.22, *p* = 0.28). Moreover, no significant correlation was found between interhemispheric functional connectivity and Conners ADHD H/I symptoms in either children with ADHD (*r* = −0.03, *p* = 0.91) or in TD controls (*r* = 0.04, *p* = 0.87).

## Discussion

Our primary finding was that finger-tapping-task-related interhemispheric connectivity modulation correlated with Conners ADHD inattentive symptoms in children with ADHD during non-dominant finger tapping (LHFT) (*r* = 0.55, *p* = 0.01) but not with H/I symptom severity (*r* = −0.09, *p* = 0.75). This association persists even when adjusting for correlations between connectivity modulation and ERD. By contrast, we had previously identified that among children with ADHD, intra-hemispheric alpha ERD correlates with ADHD H/I symptoms but not with inattentive symptoms (Luo et al., 2022). In support of our primary goal, we have evidence external validity using EEG for double dissociation between H/I and inattentive symptoms of ADHD. These results are convergent with a prior study that used resting-state fMRI to establish a double dissociation (Sanefuji et al., 2017).

Using physiological data as external validation of cognitive mechanisms is helpful in terms of validating endophenotypes and developing an ontology of cognitively biologically isolable components of complex syndromes such as ADHD (Poldrack et al., 2011), which reflects a necessary first step in the development of biomarkers that are capable of parsing this multifaceted and heterogeneous condition in both research and clinical contexts (Ewen et al., 2019). These utilitarian functions of neuroimaging may be justified even if we do not have a full understanding of the neurobiological mechanisms that drive them (Ewen et al., 2021).

The association between interhemispheric connectivity modulation and inattention symptomatology in the current sample was unexpected, as the literature around the role of interhemispheric connectivity in selective or sustained attention is sparse, both in reference to ADHD and more broadly. A large segment of the interhemispheric attention literature focuses on the integration of visual information presented to different hemifields (Akin et al., 2014; Plow et al., 2014; Mohamed et al., 2015), in which interhemispheric attention performance is either downstream of more general attentional variation or is a separate phenomenon; these perspectives do not necessarily shed light on how our connectivity findings are related to the broader set of behaviors that are reflected in the Connors ADHD Inattention scale. Banich has proposed one model of attention that could provide a framework for following up on the role of altered interhemispheric interaction in ADHD (Banich, 1998).

Separately, in prior papers (Mcauliffe et al., 2020; Luo et al., 2022), we have used these physiological findings during finger-tapping findings to evaluate contrasting mechanistic models of mirror overflow. Results to date have supported the “Ipsilateral Cortical-Spinal Tract” model that posits that mirror overflow is caused by non-decussating fibers from the motor regions of the hemisphere contralateral to the volitional movement to the opposite hand. During LHFT, this would obtain as the right hemisphere generating volitional signals to the left hand *via* decussating cortical-spinal fibers and simultaneously generating mirror-overflow-causing signals to the right hand *via* non-decussating fibers. Our results to date have not, however, been consistent with the “Transcallosal” model, which posits that signals from the right cerebral motor regions transfer through the corpus callosum to the left cerebral motor regions, where they generate further signals that flow *via* decussating cortical-spinal fibers to the right hand, generating mirror overflow. Under the assumption that our metrics of task-related functional connectivity modulation are sensitive to differences in this transcallosal communication, and consistent with prior lack of support for the Transcallosal model in the same sample, we did not find statistically significant group (ADHD vs. TD) differences in connectivity modulation. However, a couple of findings were not completely consistent with falsifying the role of transcallosal communication in the production of mirror overflow: First, given the “small to moderate” effect size of *d* = 0.45 (despite inability to reject the null), this negative finding may represent an underpowered inference and should be tested in a larger sample. Additionally, the TD group, while showing considerably less overflow than the ADHD group, did demonstrate a significant correlation between magnitude of overflow and task-related functional connectivity modulation, supporting a role of transcallosal signaling in progressive disinhibition of mirror overflow movements across child development.

Several limitations need to be considered in interpreting our primary conclusion, which is the identification of a dissociation between EEG correlates of H/I symptom severity and Inattentive symptom severity. First, a wider range of tasks, recording modalities and literature-guided analyses would be helpful in better examining biological dissociations between H/I and Inattentive symptoms, as would a wider age- and severity-range of participants. Additionally, the sample size is relatively small. While the number of participants meets the requirement of minimum sample size, a larger sample would increase statistical power. If, for example, a further study were to have greater statistical power, group differences in task-related connectivity modulation may indeed become apparent, allowing for more nuanced examination of the still high plausible role of interhemispheric communication in generating mirror overflow. Finally, given that both mirror overflow and the symptoms of ADHD are phenomena with developmentally sensitive courses, and longitudinal data will be critical to understanding how biology and phenomenology co-evolve.

## Data Availability Statement

The raw data supporting the conclusions of this article will be made available by the authors, without undue reservation.

## Ethics Statement

The studies involving human participants were reviewed and approved by the Institutional Review Board of Johns Hopkins Medicine. Written informed consent to participate in this study was provided by the participants’ legal guardian/next of kin.

## Author Contributions

YL: methodology, data analysis, and writing original draft. JA and DC: investigation, data acquisition, and data curation. SM: funding acquisition, project administration and reviewing, and editing of the manuscript. JE: conceptualization, supervision and reviewing, and editing of the manuscript. All authors contributed to the article and approved the submitted version.

## Conflict of Interest

The authors declare that the research was conducted in the absence of any commercial or financial relationships that could be construed as a potential conflict of interest.

## Publisher’s Note

All claims expressed in this article are solely those of the authors and do not necessarily represent those of their affiliated organizations, or those of the publisher, the editors and the reviewers. Any product that may be evaluated in this article, or claim that may be made by its manufacturer, is not guaranteed or endorsed by the publisher.
